# Impact of the COVID-19 pandemic and COVID vaccination campaign on imaging case volumes and medicolegal aspects

**DOI:** 10.3389/frhs.2024.1253905

**Published:** 2024-02-29

**Authors:** Caterina Battaglia, Francesco Manti, Daniela Mazzuca, Antonio Cutruzzolà, Marcello Della Corte, Fiorella Caputo, Santo Gratteri, Domenico Laganà

**Affiliations:** ^1^ Department of Experimental and Clinical Medicine, “Magna Graecia” University, Catanzaro, Italy; ^2^ Department of Surgical and Medical Sciences, University “Magna Græcia” of Catanzaro, Catanzaro, Italy

**Keywords:** COVID-19, radiology, teleradiology, artificial intelligence, vaccine, outpatient

## Abstract

**Purpose:**

The coronavirus pandemic (COVID-19) significantly impacted the global economy and health. Italy was one of the first and most affected countries. The objective of our study was to assess the impact of the pandemic and the vaccination campaign on the radiological examinations performed in a radiology department of a tertiary center in Southern Italy.

**Materials and methods:**

We analyzed weekly and retrospectively electronic medical records of case volumes performed at the Radiology Department of “Mater Domini” University Hospital of Catanzaro from March 2020 to March 2022, comparing them with the volumes in the same period of the year 2019. We considered the origin of patients (outpatient, inpatient) and the type of examinations carried out (x-ray, mammography, CT, MRI, and ultrasound). A non-parametric test (Wilcoxon Signed Rank test) was applied to evaluate the average volumes.

**Results:**

Total flows in the pandemic period from COVID-19 were lower than in the same pre-pandemic period with values of 552 (120) vs. 427 (149) median (IQR) (*p* < 0.001). The vaccination campaign allowed the resumption of the pre-vaccination pandemic with total flows 563 (113) vs. 427 (149) median (IQR) *p* < 0.001. In the post-vaccination period, the number of examinations was found to overlap with the pre-COVID period.

**Conclusion:**

The pandemic impacted the volume of radiological examinations performed, particularly with the reduction of tests in outpatients. The vaccination allowed the return to the pre-COVID period imaging case volumes.

## Introduction

The World Health Organization (WHO) declared the COVID-19 pandemic in March 2020 (1, 2). Around the world, 3,502,956 cases and 245,081 deaths have been reported as of 3 May 2020 (3). The latest WHO report of May 8, 2022, confirmed more than 514 million positive cases, with 6 million deaths (4). Italy was one of the first countries to be involved and one of the most affected (5). The effects of lockdown and the impossibility of moving from our own houses, except for emergencies, contributed to the discouragement of the examinations (6). In particular, pandemic had a significant impact on imaging case volumes in hospitals (7). Due to social distancing measures and a decrease in overall hospital activity, many imaging departments have seen a decrease in case volumes. This has been particularly true for elective procedures, which have been put on hold to prioritize urgent and emergent cases related to COVID-19. In addition to the decrease in case volumes, the pandemic has also led to changes in the composition of imaging volumes. For example, there has been an increase in the use of telemedicine and remote monitoring. Furthermore, the COVID-19 pandemic has greatly impacted the functioning of imaging departments in hospitals. It has led to a decrease in case volumes and revenue, as well as changes in the composition of imaging volumes. These effects are likely to be felt for some time, and hospitals needed to adapt and find new ways to maintain revenue and support the education of radiology residents in the face of these challenges (8). The vaccination campaign implemented in Italy allowed a decrease in infections and, above all, a reduction in mortality associated with the virus. This led to the economy's recovery and access to treatment that had been drastically slowed down during the pandemic period (9, 10). From a forensic point of view, the reduction in the volume of diagnostic tests due to poor accessibility and infectious risk may, in some cases, pose problems in the area of delay in diagnosis and medical malpractise (11). The aims of the present research were two: the first one was to evaluate the impact of containment measures, adopted during the COVID-19 pandemic, on imaging case volumes in a tertiary center of south Italy; the second one was to assess the effect of large-scale vaccination on the same volumes in this center.

## Material and methods

A retrospective analysis of the imaging examination volumes of the Radiology Department at the “Mater Domini” University Hospital of Catanzaro was performed from March 2019 to March 2022 to assess the impact of the COVID-19 pandemic. Imaging case volumes were assessed as number of exams performed weekly (from Sunday to Saturday) from March 2019 to March 2022 (156 weeks). We considered three distinct period interval: (1) the period affected by COVID-19 lockdown measurement (COVID-preVax) that went from the first week of March 2020 to the last week of February 2021(52 weeks); (2) The period in which we expected a significant impact from COVID vaccination campaign (COVID-postVax), from the first week of March 2021 to the last week of February 2022 (52 weeks); (3) The reference period before COVID pandemic occurred (pre-COVID), that went from the first week of March 2019 to the last week of February 2020 (52 weeks). Infact, the first cases of COVID-19 were reported in Italy as of March 2020. We chose to start considering the COVID-postVax period as of March 2021, as the vaccination campaign began on January 2021 at our hospital, and by assuming a protective effect of the vaccine after the second shot (one month later). We analyzed and compared the case volumes between the three time intervals as total number of exams performed weekly, and also as number of exams divided by the patient's type (inpatient, outpatient) and by the type of imaging used (x-rays, mammography exams, Computed Tomography, Magnetic Resonance, Ultrasound). Data of the case volumes were traced separately to demonstrate the trend of weekly volumes of imaging cases in 2020. The analysis in imaging volumes between time periods were matching the same weeks, in order to consider the monthly or seasonal variation within the same calendar year. No significant changes in the imaging devices or staff during the study period occurred in our radiology department that could have impacted the number of radiological procedures. The normality distribution of vascular parameters collected during the study was assessed graphically and with the Shapiro–Wilk test. According to the distribution, continuous variables were expressed as median (IQR). A non-parametric test (Wilcoxon Signed Rank test) was performed, due to a skew negative distribution, by comparing the average weekly volumes of imaging cases from 2019 to 2022 for pre-COVID-19 and post-COVID-19 (COVID-preVax and COVID-post-Vax) periods. Statistical significance was considered for values of *p* < 0.05. Statistical analysis was performed using IBM SPSS Statistics for Windows, Version 25.0. Armonk, NY: IBM Corp. The study was conducted in accordance with the Declaration of Helsinki. For the first aim, our null hypothesis was that the number of weekly exams during the COVID-preVax period was not different from that of the pre-COVID period. The alternative hypothesis was that the number of weekly exams during the COVID-PrevVax period was reduced from that of the pre-COVID period. By assuming of number of exams of 400 ± 150, from our preliminary data, and considering as statistical significant a reduction of at least 12%, i.e., 48 exams (7) we calculate our sample size with the formula: n = (Z_α/2 _+ Z_β_)^2^ × 2 × σ^2^/d^2 ^= 154 where Z_α/2_ for a confidence level of 95%, α is 0.05 and the critical value is 1.96, Z_β_ for a power of 80%, β is 0.2 and the critical value is 0.84, σ^2^ is the population variance and it is 22,500, and d is the difference we would like to detect, i.e., 48. Therefore, our sample size calculation was of at least 154 exams per week. For the second aim, we assumed an alternative hypothesis where that number of exams per week during the COVID-postVax period increased of at least 12% compared to the COVID-preVax. Thefore the sample size was the same as the first aim.

## Results

**Figure 1:**
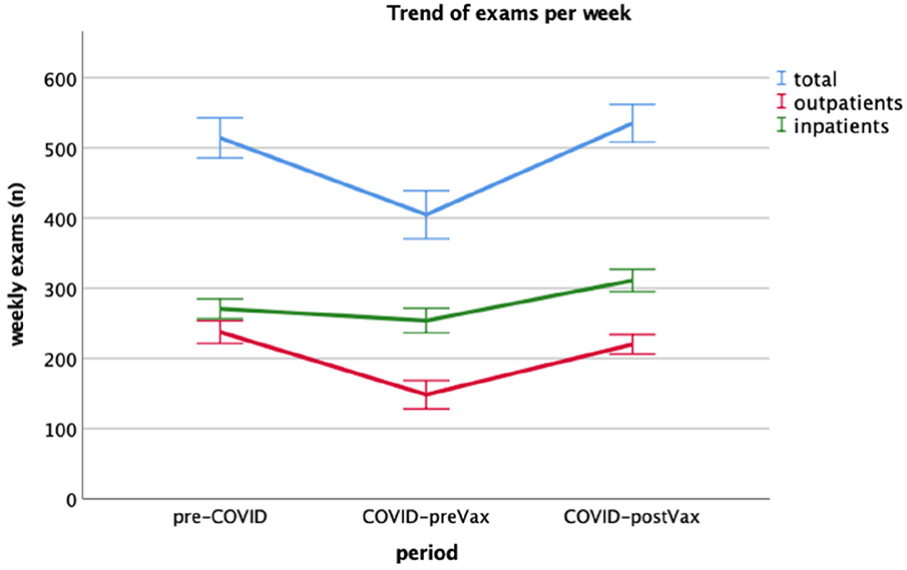
The change in the number of radiological exams divided into three categories of provenience: total (blue), outpatients (red), and inpatients (green) performed at mater Domini, Magna Græcia University of Catanzaro (UMG), in each study period: pre-COVID, COVID-preVax, and COVID-postVax.

A total of 47.787 examinations were evaluated. The total amount of radiological examinations performed during the COVID-preVax period were significantly lower than those executed during the pre-pandemic period, with 552 (120) vs. 427 (149) weekly exams [median (IQR), 95% confidence interval, *p* < 0.001]. However, the reduction of the exams performed during the COVID-preVax period compared to the pre-COVID-19 period was statistically significant only for outpatients (242 (87) vs. 171 (114), 95% confidence interval, *p* < 0.001), but not for inpatients, (282 (60) vs. 268(87), *p* = 0.42). The vaccination campaign allowed a rise in flows compared to the COVID-preVax period, with 563 (113) vs. 427 (149) exams, 95% confidence interval, *p* < 0.001. The increase in flows was statistically significant in both inpatient and outpatient patients, with 333 (73) vs. 268 (87) COVID-preVax median (IQR), 95% confidence interval, *p* < 0.001 and 229 (62) vs. 171 (114) median (IQR), 95% confidence interval, *p* < 0.001 (Figure 1).

**Figure 2:**
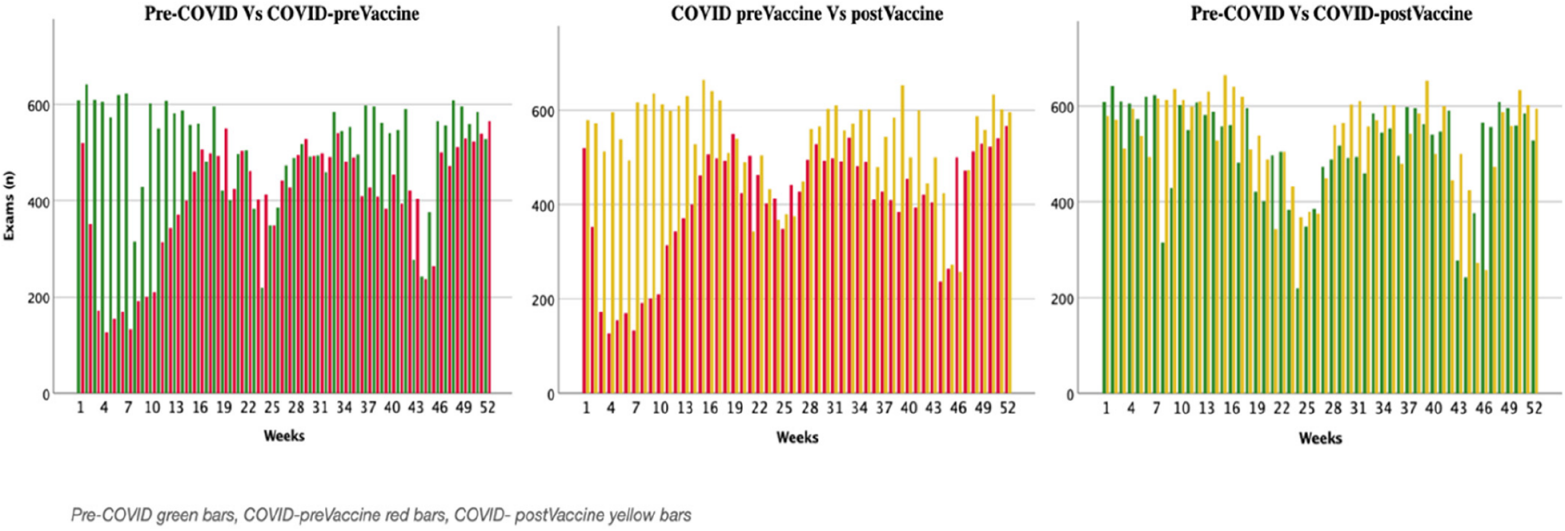
Representation of the change in the number of radiological exams divided into three periods: pre-COVID (green bars), COVID-preVax (red bars), and COVID-postVax (yellow bars) performed at mater Domini, Magna Græcia University of Catanzaro (UMG).

In the COVID-postVax period, a resumption overlapped with the pre-COVID period (Figure 2).

Concerning data divided by method, ultrasound, conventional radiology, breast imaging, Computed tomography, and MRI, all suffered a statistically significant reduction. Table 1 compares the post-COVID 19 and pre-COVID-19 period mean weekly imaging case volumes for the imaging modality types stratified by patient service locations with statistically significant values.

**Table 1:** Data was divided by radiological examination method with weekly acquired values among pre-COVID, COVID-preVax, and COVID-postVax periods.

Radiological examination	Pre-COVID 19	COVID-preVax	COVID-postVax
US	138 (37)	100 (49)*	133 (32)^#,†^
x-ray	235 (70)	188 (86)*	248 (53)^#^
Senology	37 (24)	17 (20)*	42 (27)^#^
CT	99 (19)	89 (28)*	105 (27)^#^
MRI	30 (6)	27 (16)*	33 (8)^#,†^

* *p* < 0.05 COVID-preVax vs. pre-COVID.

^#^ *p* < 0.05 COVID-postVax vs. COVID-preVax.

^†^ *p* < 0.05 COVID-postVax vs. pre-COVID.

## Discussion

The COVID-19 pandemic showed unprecedented and unpredictable circumstances for radiology practices. The impact observed in all over the world was severe in terms of health care (12). Our study showed how this devastating situation has impacted on imaging case volumes in a tertiary hospital center in South Italy. In particular, we observed a reduction of 23% during the COVID-19 pandemic compared with the pre-COVID period, mainly due to the decline in the number of exams performed on outpatients. Our findings align with many other studies, which have demonstrated that radiology practices decreased by up to 40%–50% in imaging volumes, depending on the location of work and the severity of the COVID-19 pandemic in every region (7, 13). In our center, the reduction of radiologic examinations was probably driven by the limited access to the radiology department by chronic patients lacking our center of an emergency unit. For the first time to our knowledge, we have observed a recovery in the imaging case volumes after the vaccination campaign compared to the pre-vaccination period (+32%), with a return to the volumes registered in the pre-pandemic period. Vaccines have been a fundamental tool for reducing mortality and morbidity of COVID patients (14). Our findings demonstrated that the vaccines have also allowed the restart of public health systems, thus guaranteeing access to care for patients again. This reduction of examinations caused diagnostic delay with associated medical-legal disputes. Especially in the case of specific pathologies, such as cancer, where timely diagnosis significantly impacts the prognosis (15, 16). The lack of access to radiological examinations could configure what is defined as a loss of chances (17–19). The reduction of imaging case volume was justified by the absolute need to protect healthcare personnel and patients from infectious risk. For avoiding this issues technology and science plaied a significant part in particular innovative tools as teleradiology and artificial intelligence (AI) were implemented and for a better care for affected patients all over the world (20, 21). AI is made to act and think like a human brain, automating many tasks by imitating its thought processes. In preparation for COVID-19's eventual cross-country accessibility, machine learning (ML) and deep learning (DL) techniques have been utilized to track typical behavior using open data sources from real-time applications. These techniques could forecast the immediate future and aid in minimizing the negative impacts of COVID-19 (22). For example in a study of Alaiad et al. they applied Deep Learning, called Resnet101, on CT images in hospitals to complement the work, giving them high confidence in the uses of AI, especially deep learning on images in their hospital (23, 24). Regarding teleradiology although it offers the benefits of saving patient's time and safety, it is necessary to make known limitations, including the absence of physical examinations, the possibility of transmission failure and risks of violations of privacy. In term of regulation, in Italy, the first provision dedicated to the implementation of Telemedicine services is represented by the Agreement between the Government and the Regions on the document containing “Telemedicine-National Guidelines”. guidelines are intended to respond to the need to standardize the experimental initiatives activated (25). According to a review by Solimini et al., the ethical and legal issues related to telemedicine services still need specific rules for the application to guarantee equitable access, quality of care and respect of patient privacy, data protection, and data protection sustainable costs (26). In the future the use of telemedicine and AI in clinical will be used in common clinical practice (27, 28).

## Limitations and further studies

The study is limited by its retrospective design. The economic effects of this decline in imaging case volumes and the effects on the radiology residency program were not investigated. These limitations could serve as an avenue for future research.

## Conclusion

The pandemic had a negative impact on the volume of radiological examinations performed, notably by limiting access to care for outpatients. According to this report, the total imaging volume across all patient care locations and imaging modalities decreased by 23% during the COVID-19 pandemic. The vaccination campaign allowed the return to the imaging flow of the pre-COVID period. Effective strategies must be adopted to avoid the mentioned effect on the healthcare system in future pandemics.

## Data Availability

The original contributions presented in the study are included in the article/Supplementary Material, further inquiries can be directed to the corresponding author.
